# Characterising depression trajectories in young people at high familial risk of depression

**DOI:** 10.1016/j.jad.2023.05.063

**Published:** 2023-09-15

**Authors:** Bryony Weavers, Lucy Riglin, Joanna Martin, Richard Anney, Stephan Collishaw, Jon Heron, Ajay Thapar, Anita Thapar, Frances Rice

**Affiliations:** a Wolfson Centre for Young People's Mental Health, Division of Psychological Medicine and Clinical Neurosciences, Cardiff University, Wales, UK; b Centre for Neuropsychiatric Genetics and Genomics, Division of Psychological Medicine and Clinical Neurosciences, Cardiff University, Wales, UK; c Population Health Sciences, Bristol Medical School, University of Bristol, Bristol, Gloucestershire, UK

**Keywords:** Depressive disorder, High risk, Longitudinal, Trajectories, Developmental

## Abstract

**Background:**

Parental depression is a common and potent risk factor for depression in offspring. However, the developmental course of depression from childhood to early-adulthood has not been characterized in this high-risk group.

**Methods:**

Using longitudinal data from 337 young people who had a parent with a history of recurrent major depressive disorder (MDD), we characterized trajectories of broadly defined depressive disorder using latent class growth analysis. We used clinical descriptions to further characterise trajectory classes.

**Results:**

Two trajectory classes were identified: childhood-emerging (25 %) and adulthood-emerging (75 %). The childhood-emerging class showed high rates of depressive disorder from age 12.5, which persisted through the study period. The adulthood-emerging class showed low rates of depressive disorder until age 26. Individual factors (IQ and ADHD symptoms) and parent depression severity (comorbidity, persistence and impairment) differentiated the classes but there were no differences in family history score or polygenic scores associated with psychiatric disorder. Clinical descriptions indicated functional impairment in both classes, but more severe symptomatology and impairment in the childhood-emerging class.

**Limitations:**

Attrition particularly affected participation in young adulthood. Factors associated with attrition were low family income, single parent household status and low parental education.

**Conclusions:**

The developmental course of depressive disorder in children of depressed parents is variable. When followed up to adult life, most individuals exhibited some functional impairment. An earlier age-of-onset was associated with a more persistent and impairing course of depression. Access to effective prevention strategies is particularly warranted for at-risk young people showing early-onsetting and persistent depressive symptoms.

## Highlights

• Adulthood-emerging and childhood-emerging trajectory classes were identified.
• The childhood-emerging class showed a severe and persistent course of depression.
• Most individuals in both classes experienced some functional impairment.

## 1 Introduction

Major depressive disorder (MDD) is a leading cause of global disability (Vos et al., 2012) and is projected to result in the highest burden of disease globally by 2030 (Malhi and Mann, 2018). One of the most common, potent risk factors for MDD is having a parent with a history of depression (Weissman et al., 2016). Descriptive studies of youth and adult depression show that family history of depression in a parent is associated with an earlier age of depression onset, a more persistent course of disorder, greater comorbidities and higher rates of psychosocial impairments (Lieb et al., 2002; Nierenberg et al., 2007; Weissman et al., 2006).

Clinical family studies of depression using diagnostic instruments to assess mental health in the offspring of depressed parents have reported 4-fold elevated rates of MDD, 3-fold risk of suicide attempts, high rates of psychiatric hospitalization (40 % compared to 1 %) and overall impaired functioning (Weissman et al., 1999, Weissman et al., 2016), compared to control groups of offspring with non-depressed parents. Family studies also suggest that exposure to current parental depression, increased severity of parental depressive episodes and the burden of co-occurring psychopathology in parents is associated with a heightened risk of depression and other psychiatric conditions in offspring (Brophy et al., 2021; Mars et al., 2012; Sellers et al., 2013).

Depression encompasses a spectrum ranging from sub-threshold symptoms to a diagnosis of MDD. Such variation is important to consider, as episodic mood fluctuations can be part of typical development. However, identifying when symptoms depart from a typical trajectory is critical in identifying individuals at risk of developing depressive disorder (Thapar and Riglin, 2020). Describing patterns of depression trajectory classes over time is one way of capturing heterogeneity in the developmental course of depression. Such designs have been used extensively to study depression in young people in the general population (Musliner et al., 2016; Schubert et al., 2017), but not with a high-risk sample.

In the general population, trajectory studies of self-reported depressive symptoms have found multiple classes, most often 3 or 4, that varied in severity, stability and age-of-onset, with the majority of participants showing consistently low symptoms and a minority showing persistent symptoms (Musliner et al., 2016). Risk factors associated with a trajectory of elevated depressive symptoms have included female sex, neurodevelopmental traits and genetic liability to psychiatric conditions indexed by polygenic scores (PGS). However, it is not known whether the offspring of depressed parents show similarly diverse depression developmental trajectories, or whether risk factors that differentiate trajectory classes in the general population also apply to the offspring of depressed parents.

Many developmental trajectory studies in the general population do not consider clinically elevated symptoms or functional impairment, and instead look at continuous self-reported symptoms when examining depressive symptomatology. Indeed, the majority of previous studies relied on self-reported depressive symptoms across the full range of symptomatology and include no measure of impairment (Schubert et al., 2017). We used a broad definition of depressive disorder that encompassed one core symptom of MDD, one other MDD symptom and associated impairment. This study aimed to characterize the developmental trajectories of broadly defined depressive disorder in a sample of young people with a parent with a history of recurrent MDD according to DSM-IV (American Psychiatric Association, 1994). Next, we examined the associations of these developmental trajectory classes with factors identified from previous research in the general population as differentiating ‘persistently low depressive symptom’ classes from ‘elevated depressive symptom’ classes. In the current study, we included female sex, attention deficit hyperactivity disorder (ADHD) symptoms, IQ, degree of familial loading for depression and genetic liability to psychiatric and neurodevelopmental conditions, as indexed by PGS for MDD, ADHD, schizophrenia and bipolar disorder (Rice et al., 2019; Schubert et al., 2017; Shore et al., 2018). We also examined parental depression severity, parental comorbidities and co-parent depression, which have been identified as risk factors for offspring depression in family studies (Brophy et al., 2021; Dierker et al., 1999; Mars et al., 2012; Merikangas et al., 1988; Sellers et al., 2013). A final aim involved using deeply phenotyped data to derive brief clinical descriptions of selected individuals to provide a richer narrative of the trajectory classes. Based on previous literature on general population samples we expected to find more than one trajectory class (Musliner et al., 2016; Schubert et al., 2017). Given the elevated level of risk in this population we expected a smaller proportion of individuals to fall into a ‘persistently low depression’ group and more to fall into an ‘elevated high depression’ group, compared to previously published general population samples (Lieb et al., 2002). We also expected that predictors previously associated with an elevated course of depressive symptomatology in the general population would extend to this sample.

## 2 Methods

### 2.1 Participants

The Early Prediction of Adolescent Depression (EPAD) study is a four-wave prospective longitudinal study of 337 families. Parents were recruited if they had a history of recurrent depression (characterized as at least two DSM-IV MDD episodes confirmed at interview) and lived with a child (age 9–17 years) to whom they were biologically related. If more than one child was eligible, the youngest of those eligible and willing to participate was selected (Mars et al., 2012). At the baseline assessment, children were aged 9–17 years (mean age 12.4) and at the fourth wave they were young adults aged 18–28 years (mean age 23.4). Ethical approval was granted by the Multi-Centre Research Ethics Committee for Wales and from the School of Medicine Ethics Committee, Cardiff University. Family response rates for each wave can be found in Supplementary Fig. S1. One family did not complete an interview at any wave (only self-reported questionnaires) and were therefore excluded from the analysis, such that 336 families were included (58.5 % female offspring, 93.5 % female parents).

### 2.2 Procedures

Assessments were undertaken on four occasions (waves) between April 2007 and September 2020 (Supplementary Text). Families were recruited at baseline primarily from general practices across South Wales, UK (78 %), from a previous community study of recurrent unipolar depression (19 %), and from additional sources such as posters within primary care centers (3 %). After participants were screened for eligibility, interviews were scheduled. Written informed consent (and assent for children under age 16) was gained from each participant at each wave. Trained research assistants assessed the parent and young person separately using semi-structured interviews. Parents and young people also completed questionnaires that were sent via post prior to the assessment. Visits primarily took place at the participant's home, but a small number of assessments were undertaken over the telephone or video call. The parent and child assessments were completed independently, and in most instances in separate rooms and by separate researchers. The average length of follow up between the first and second wave was 16 months, between the second and third wave 13 months, and between the third and fourth wave 8 years (See Supplementary Fig. S1).

### 2.3 Design

A prospective longitudinal study with four assessments over a 13-year period. We used an accelerated longitudinal design to derive trajectories and modelled these by age. Sample size for each young person age following restructuring the data into an accelerated design can be found in Supplementary Table S1.

### 2.4 Measures

#### 2.4.1 Young person's depression

The primary measure of depression in young people was derived using the Child and Adolescent Psychiatric Assessment (CAPA) (Angold et al., 1995) at the first three waves and its adult extension, the Young Adult Psychiatric Assessment (YAPA) (Angold et al., 1999a), at the fourth wave. The CAPA and YAPA are semi-structured diagnostic interviews and were used to assess depressive disorder. At each assessment, the CAPA/YAPA was completed with young people and parents about the young person's symptoms over the preceding 3-months. A binary ‘depression’ variable was created to represent a broad depressive disorder phenotype. This was defined as one core symptom of DSM-IV MDD (2 weeks of low mood, loss of interest or pleasure, or irritability) in addition to at least one other symptom of MDD, as well as associated impairment (measured as part of the CAPA/YAPA). This definition has been used in previous research (Rawal et al., 2013) and there is good evidence that depression can be viewed in this way given that symptoms falling below the diagnostic threshold are associated with functional impairment and predict later episodes of MDD (Angold et al., 1999b; Thapar et al., 2022). Symptoms were considered present if either the parent or young person endorsed the symptom. Both interview schedules required the interviewer to record detailed clinical descriptions.

#### 2.4.2 Validators of trajectory class

To gain an understanding of the meaning of the trajectory classes we examined associations with several ‘validator’ variables. These were indicators of psychopathology (depression and anxiety) and functional impairment, at baseline and early adulthood follow-up. Further details of these measures can be found in the Supplementary Text and they are described briefly below.

DSM-IV MDD diagnoses were derived using diagnostic information from the CAPA/YAPA interviews, to assess the clinical validity of the depression trajectory classes, given our focus on broadly defined depressive disorder. All diagnoses were reviewed by two experienced clinicians and made by consensus. Depressive symptoms were assessed using The Mood and Feelings Questionnaire (MFQ) (Costello and Angold, 1988) total scores (self- and parent-reported separately; score range 0–66 and 0–68 respectively), to quantify the levels of elevated but sub-diagnostic threshold depressive symptomatology. The MFQ is a well-validated screener for depression in children and young adults (Eyre et al., 2021; Wood et al., 1995). We assessed anxiety disorders given that family studies have found high rates of early-onset anxiety in offspring at high familial risk of depression which often precede the onset of depression (Weissman et al., 1997, Weissman et al., 2005). Generalised anxiety disorder, separation anxiety, social anxiety, agoraphobia, panic disorder and anxiety disorder NOS were assessed using the CAPA/YAPA, derived using the same method as MDD diagnoses. Functional impairment of four domains (home life, friendships, studies or work and leisure activities) was assessed using the self-reported Strengths and Difficulties Questionnaire (SDQ) impact supplement (Goodman, 1999). A score of 2 or more (range 0–10) indicated functional impairment.

#### 2.4.3 Predictors of trajectory class

All predictors were measured at baseline. Young person sex was measured using parent-reported biological sex (0 = male, 1 = female). Young person ADHD symptoms were derived by summing the 18 binary DSM-IV symptoms from the parent-reported ADHD section of the CAPA (Angold et al., 1995). Young person IQ was assessed using the full Wechsler Intelligence Scale for Children (WISC-IV) (Wechsler and Kodama, 1949).

A weighted family history depression score for the young person was derived using the proportion of family members with a history of depression weighted by relatedness of the young person (first or second-degree relatives) (Milne et al., 2009). Polygenic scores for each individual were calculated for 10 *P*-value thresholds from genome-wide association studies (GWAS) for ADHD, MDD, schizophrenia, and bipolar disorder (Demontis et al., 2019; Mullins et al., 2021; The Schizophrenia Working Group of the Psychiatric Genomics Consortium et al., 2020; Wray et al., 2018). Using PGS principal component analysis (PCA) (Coombes et al., 2020), a composite score, specifically the first-principal component of the combined-by-GWAS score was then used in the model. PCs were also added into the model as covariates (see Supplementary text for further details).

The proportion of assessment points during which the index parent met current DSM-IV MDD criteria was derived using the depression section of the Schedules for Clinical Assessment of Neuropsychiatry (SCAN) (Wing et al., 1990). The presence of a severely impairing depressive disorder was defined by a Global Assessment of Functioning (GAF) score (American Psychiatric Association, 1994) of <50 for the worst ever depressive episode, measured at baseline. A total score (range 0 to 3) of co-occurring problems (anxiety, antisocial behaviour and harmful drinking), in addition to depression at baseline, was derived, as used previously (Sellers et al., 2013). Co-parent depression score was derived by summing the nine depressive items (based on DSM-IV MDD criteria) of the Patient Health Questionnaire (PHQ) (Kroenke et al., 2001), each coded 0–3 (range 0–27).

#### 2.4.4 Clinical descriptions

Clinical descriptions were produced at each assessment point by researchers who interviewed the families, summarising current symptoms and significant life events. These were written separately for self-reported child symptoms, self-reported parent symptoms and parent-reported child symptoms. In this study, individuals were selected using purposeful sampling where individuals with the highest probability of being assigned to each of the trajectory classes were chosen in order of probability. We made use of descriptions of symptomatology to supplement the quantitative analysis and further characterise the depression trajectories.

### 2.5 Statistical analysis

We treated the data as an accelerated longitudinal design (which was possible due to the range of ages at recruitment) and fitted models of depression (broadly defined depressive disorder) by age (range 9–28 years). This design made the assumption of exchangeability, such that there were no substantial effects of wave on trajectory class assignment. We examined this by testing age at each wave as a correlate of class membership and testing the effects and interaction of age and wave on depressive disorder (Supplementary Tables S2 and S3). We found no evidence for effects of this nature.

We used latent class growth analysis (LCGA) (B. Muthén and Muthén, 2000) in MPlus version 8.8 (L. K. Muthén and Muthén, 2017) with full information maximum likelihood (FIML) to derive trajectory classes. We first compared linear, quadratic and piecewise models to test which model provided the best fit to the data. Model selection was informed by overall model fit indices including sample size-adjusted Bayesian information criteria (SABIC), loglikelihood (LL), Vuong-Lo-Mendell-Rubin Likelihood Ratio Test (VLMR-LRT) and Bootstrap Likelihood Ratio Test (BLRT). FIML, which makes a missing-at-random assumption, permitted partially incomplete data to be included. To estimate the proportion or mean of phenotypic traits shown in each class for each of the considered variables and for equality tests of means across classes, the BCH approach (Asparouhov and Muthén, 2019), implemented in Mplus was used.

#### 2.5.1 Sensitivity checks

Irritability was included as a core symptom for depressive disorder at all ages. In the DSM-IV, irritability is a core MDD symptom for children and adolescents but not in adults. As a sensitivity analysis irritability was excluded when defining broadly defined depressive disorder. The new means and standard deviations for depressive disorder and subsequent trajectory classes were derived. When deriving broadly defined depressive disorder, symptoms were considered present if either the parent or child reported them as present. This is recommended in clinical practice when assessing young people (De Reyes and Kazdin, 2005). We included correlations between parent and self-reported symptoms and ran the trajectory class analysis using self-reported data in adulthood as a sensitivity analysis.

## 3 Results

### 3.1 Depression trajectories

**Fig. 1:**
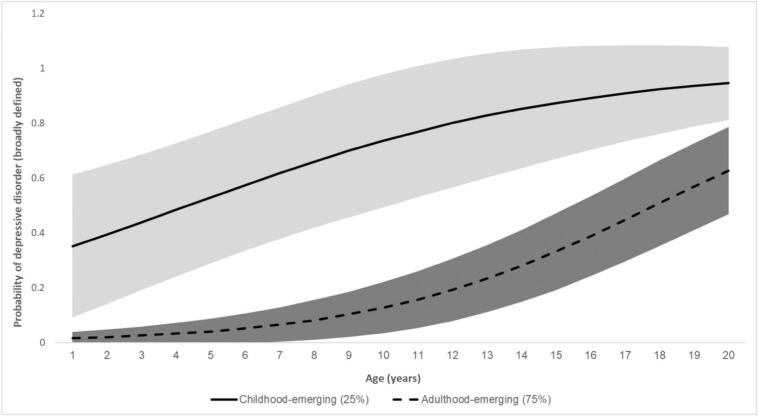
Trajectory classes. This figure shows the two trajectory classes and 95 % confidence interval bands: adulthood-emerging (75.4 %, N ≈ 253) and childhood-emerging (24.6 %, N ≈ 83). The Ns are approximate due to the probabilistic nature of LCGA whereby it is not possible to think of class size in terms of a whole number of people.

A single linear trajectory was modelled. Other models including a quadratic function or additional slopes to capture different developmental periods did not show improved model fit and were less parsimonious (Supplementary Table S4). We modelled 1-to-3-class solutions. Model fit improved from 1-class to the 2-class solution, and although LL improved from the 2-class to the 3-class solution, other model fit parameters, such as SABIC, worsened and the small sample size of the smallest class (10.5 %, n ≈ 35) indicated that the 2-class solution was the most appropriate and best fitting model (Table 1). Fig. 1 shows the two trajectory classes: childhood-emerging (24.6 %, N ≈ 83) and adulthood-emerging (75.4 %, N ≈ 253). It also shows 95 % confidence interval bands, which did not overlap for the classes. Depression was considered clinically relevant when probability of meeting broadly defined depressive disorder equalled or exceeded 0.5. This was approximately age 12.5 for the childhood-emerging class and age 26 for the adulthood-emerging class; thus the classes were named based on the developmental period during which depressive disorder emerged. The childhood-emerging class showed a high probability of depressive disorder from a young age, which increased throughout adolescence and reached 0.95 at age 28. In contrast, the adulthood-emerging class showed low probabilities of depressive disorder throughout childhood and adolescence, which increased to a clinically significant level in early adulthood (Fig. 1).

**Table 1:** Model fit indices for selecting the best fitting model.

Number of classes	1	2	3
SABIC	984.043	914.882	917.550
LL	−489.376	−450.829	−448.195
Number of parameters	2	5	8
Entropy	–	0.623	0.493
Smallest class	–	24.6 % (*n* = 83)	10.5 % (*n* = 35)
Average latent class probabilities for most likely class membership	–	Class 1: 0.92 Class 2: 0.81	Class 1: 0.65 Class 2: 0.78 Class 3: 0.74
VLMR-LRT *p* value	–	<0.0001	0.0652
BLRT *p* value	–	<0.0001	0.3333

SABIC = sample size-adjusted Bayesian information criteria, LL = loglikelihood, VLMR-LRT = Vuong-Lo-Mendell-Rubin Likelihood Ratio Test and BLRT = Bootstrap Likelihood Ratio Test. The smallest class was based on the estimated model.

Table 2 describes associations of trajectory class with validator variables which were measures of depression, anxiety and functional impairment at baseline and early-adulthood follow-up. Consistent with Fig. 1, higher rates of MDD and depressive symptoms were seen in the childhood-emerging class compared to the adulthood-emerging class at baseline and follow-up. Anxiety was also more common in the childhood-emerging than adulthood-emerging class both at baseline and follow-up. In terms of functional impairment, both classes showed significant impairment at baseline and follow-up, but this was higher in the childhood-emerging class. In summary, validation of the trajectory classes showed that the childhood-emerging class had consistently higher rates of MDD, depressive symptoms, anxiety disorders and functional impairment compared to the adulthood-emerging class at both baseline and in early-adulthood. Rates and scores of depression, anxiety and functional impairment tended to increase in both classes over time. The exception to this was for the parent-reported child depressive symptom score in the childhood-emerging class but this remained high in early adulthood. Both classes showed evidence of functional impairment at baseline and follow-up as indicated by above threshold scores on the SDQ impact supplement.

**Table 2:** Tests of association of validator variables (depression, anxiety and functional impairment) with trajectory class.

	Adulthood-emerging depression, 75.4 % Percentage or mean (S.E)	Childhood-emerging depression, 24.6 % Percentage or mean (S.E)	Chi-square (p value)
*Baseline (wave 1)*			
MDD diagnosis	0.1 % (0.008)	14.3 % (0.046)	7.93 (0.005)
Self-reported MFQ score	11.76 (0.862)	24.53 (2.276)	23.51 (<0.001)
Parent-reported MFQ score	8.06 (0.642)	22.20 (1.955)	40.94 (<0.001)
Any anxiety disorder	3.2 % (0.017)	32.7 % (0.064)	16.86 (<0.001)
Functional impairment score	4.04 (0.284)	6.83 (0.545)	16.88 (<0.001)

Early adult follow-up assessment (wave 4)			
MDD diagnosis	6.6 % (0.029)	48.5 % (0.106)	12.84 (<0.001)
Self-reported MFQ score	14.37 (1.330)	32.02 (3.76)	17.44 (<0.001)
Parent-reported MFQ score	9.34 (1.165)	21.37 (4.134)	6.85 (0.009)
Any anxiety disorder	17.4 % (0.041)	46.6 % (0.120)	4.53 (0.033)
Functional impairment score	5.46 (0.423)	7.83 (0.963)	4.24 (0.039)

Scores of 27 or higher in the self-reported MFQ may indicate the presence of depression. Scores of 21 or higher in the parent reported MFQ may indicate the presence of depression. For the functional impairment score, a score of 0 = normal, 1 = borderline and 2+ = abnormal. Any anxiety disorder excluded specific phobia at both wave 1 and wave 4.

### 3.2 Sensitivity analyses

The proportion of individuals meeting broadly defined depressive disorder when excluding irritability was similar to that when including the symptom at all four assessment points (Supplementary Table S5). Similar trajectory classes were derived when irritability was excluded as a symptom (Supplementary Fig. S2). Correlations between parent- reported and self- reported symptoms showed moderate and consistent correlations at all assessment waves (range 0.350–0.499) (Supplementary Table S6), which were comparable to previous studies (De Reyes and Kazdin, 2005), and a very similar two class model best fitted the data when depression was self-reported only in adulthood (Supplementary Table S7). We report the combined results due to the increased sample size.

#### 3.2.1 Tests of association of predictor variables with trajectory class

We tested the association of predictors with trajectory class. These predictors were selected based on previous literature showing that they differentiated depressive trajectory classes in the general population and/or were identified as risk factors for offspring depression in high familial risk studies (Table 3).

**Table 3:** Tests of association of predictor variables with trajectory class.

	Adulthood-emerging depression, 75.4 % Percentage or mean (S.E)	Childhood-emerging depression, 24.6 % Percentage or mean (S.E)	Chi-square (p value)
Female sex	55.2 % (0.035)	67.8 % (0.069)	2.19 (0.139)
ADHD symptom score	1.09 (0.175)	2.49 (0.537)	5.22 (0.022)
IQ	96.81 (0.913)	89.16 (1.660)	13.38 (<0.001)
Family history depression score	0.27 (0.007)	0.28 (0.018)	0.57 (0.450)
MDD polygenic score	−0.11 (0.076)	0.13 (0.163)	1.50 (0.221)
ADHD polygenic score	0.02 (0.081)	0.22 (0.167)	0.97 (0.324)
Schizophrenia polygenic score	−1.2 (0.076)	−0.04 (0.162)	0.18 (0.674)
Bipolar disorder polygenic score	−0.08 (0.075)	0.08 (0.153)	0.70 (0.402)
Proportion of waves parent was depressed	14.6 % (0.027)	42.9 % (0.075)	10.65 (0.001)
Parent GAF score ever <50	56.8 % (0.034)	73.8 % (0.066)	4.23 (0.040)
Number of parent comorbidities	0.52 (0.054)	0.82 (0.119)	4.34 (0.037)
Co-parent PHQ depressive symptom score	2.77 (0.305)	3.71 (1.149)	0.55 (0.458)

PHQ scores of 0–4 indicate none or minimal symptoms, 5–9 indicate mild depression, 10–14 indicate moderate depression, 15–19 indicate moderately severe depression and 20–27 indicate severe depression.

Female sex was preponderant in both the childhood-emerging (68 %) and the adulthood-emerging (55 %) class, but it did not differentiate the classes (χ^2^ = 2.19, *p* = .139). The childhood-emerging class was associated with higher ADHD traits (χ^2^ = 5.22, *p* = .022) and a lower IQ score (χ^2^ = 13.38, *p* < .001) compared to the adulthood-emerging class.

Both classes had similar family history depression scores (χ^2^ = 0.57, *p* = .450). Although PGS were higher in the childhood-emerging class, the were no differences in the analyses of PGS for MDD (χ^2^ = 1.50, *p* = .221), ADHD (χ^2^ = 0.97, *p* = .324), schizophrenia (χ^2^ = 0.18, *p* = .674) and bipolar disorder (χ^2^ = 0.70, *p* = .402).

In terms of parental depression severity, 72 % of the study parents in the childhood-emerging class had experienced a severely impairing depressive episode in their lifetime, compared to 57 % in the adulthood-emerging class (χ^2^ = 4.23, *p* = .040). Study parents in the childhood-emerging class were also depressed for a greater proportion of study assessment points compared to the adulthood-emerging class: 43 % versus 15 % respectively (χ^2^ = 10.65, *p* = .001), indicating that young people in the childhood-emerging class had been exposed to a greater number of parental depressive episodes. In addition, parent comorbidities were higher in the childhood-emerging class compared with the adulthood-emerging class (χ^2^ = 4.34, *p* = .037). There were no differences between the classes in terms of co-parent depression symptom scores (χ^2^ = 0.55, *p* = .458).

#### 3.2.2 Clinical descriptions

The selection process for the clinical exemplars can be found in the Supplementary Text. Four individuals, A1–4, (one man, two women, and one non-binary individual) were sampled from the adulthood-emerging class (probability of being allocated to this class: 0.998–1.000). Five individuals, B1–5, (three men, two women), were sampled from the childhood-emerging class (probability of being allocated to this class: 0.998–0.999). Demographic details, probability of class membership and information about the informants is in Table 4. We summarise the clinical descriptions below.

**Table 4:** Characteristics of individuals selected as exemplars of each trajectory class.

ID	Probability of class membership	Biological sex (gender if different)	Age at the first wave (years)	Informants
Adulthood-emerging depression				
A1	0.999	Female	16	Both parent and child completed interviews and questionnaires at waves 1, 2, 3 and 4.
A2	0.998	Male	16	Both parent and child completed interviews and questionnaires at waves 1, 2, 3 and 4.
A3	0.998	Male	16	Both parent and child completed interviews and questionnaires at waves 1, 2, 3 and 4.
A4	0.998	Male	16	Both parent and child completed interviews and questionnaires at waves 1, 2, 3 and 4. In addition, a co-parent provided questionnaires at waves 1, 2 and 3.
A5	0.998	Female	15	Both parent and child completed interviews and questionnaires at waves 1, 2, 3 and 4.

Childhood-emerging depression				
B1	1.000	Female (non-binary)	10	Parent completed interview and questionnaires at waves 1, 2, 3 and 4. The child completed interview and questionnaires at wave 1, refused wave 2 and 3 due to social anxiety, and completed interview at wave 4.
B2	0.998	Female	13	Both parent and child completed interviews and questionnaires at waves 1, 2, 3 and 4.
B3	0.998	Female	12	Both parent and child completed interviews and questionnaires at waves 1, 2 and 3. The parent completed wave 4 interview and questionnaires, but the young person refused to take part at all at this wave.
B4	0.998	Male	9	Both parent and child completed interviews and questionnaires at waves 1, 2, 3 and 4.

##### 3.2.2.1 Childhood-emerging class

Individual A1 experienced a range of severe difficulties from childhood. They stopped attending school and were in contact with many specialist services by early adolescence. Their difficulties worsened throughout adolescence, and they were admitted as an inpatient to a Child and Adolescent Mental Health Services (CAMHS) ward. In early adulthood, they became bedbound due to chronic physical conditions. They reported several suicide attempts and continuous intrusive suicidal thoughts in early adulthood. At the final assessment they were being cared for by family at home.

Individual A2 reported severe depressive symptoms, social anxiety, sleep difficulties and restricting her diet and vomiting in early adolescence. She was in contact with CAMHS at this time. Throughout adolescence, her difficulties persisted and she reported self-harming. She stopped attending mainstream school and completed exams in a specialist centre. In early adulthood, she reported depressive symptoms, anxiety and frequent panic attacks. She had received diagnoses of autism and chronic fatigue syndrome and was in contact with mental health services. At the final assessment she was living independently but unable to work due to her heath conditions.

Individual A3 reported separation anxiety due to a challenging home life prior to the study period. In childhood, she reported sleep problems, low mood and social anxiety, as well as some symptoms of derealization. In adolescence, her low mood and anxiety persisted and she reported compulsive lying. A traumatic event at home worsened her anxiety. In early adulthood, she did not take part in the final assessment. Her mother reported she experienced anxiety symptoms following an incident at work. She had completed a degree, was in a full-time job and living independently.

Individual A4 reported depressive symptoms, including suicidal thoughts in childhood, as well as sleep problems and anxiety. In early adolescence he was seen by CAMHS. During adolescence, his depressive symptoms and sleep problems persisted, and his anxiety worsened. In adulthood, his worries continued to impair daily functioning. He dropped out of college and at the final assessment had been signed off work due to depression.

##### 3.2.2.2 Adulthood-emerging class

Individual B1 experienced significant depressive symptoms during childhood following bullying at school, however these had mostly desisted by adolescence apart from some mild low mood and irritability. She did not attend university but was employed continuously. In early adulthood she reported some depressive symptoms and difficulties socialising. She was living independently and in full-time employment.

Individual B2 reported no symptoms throughout the study period. He attended university and in early adulthood was in full-time employment, living independently and reported being in a steady relationship.

Individual B3 reported some weight fluctuations and aches and pains during adolescence and occasionally skipped school. He did poorly in secondary school exams but later attended university. In early adulthood he had a full-time job, steady relationship and was living independently.

Individual B4 reported occasional behavioural symptoms during adolescence. He attended university and in early-adulthood was working full-time and living independently.

Individual B5 reported hyperactive and inattentive traits in childhood that persisted into adult life. During adolescence she reported excessive worry, low mood and sleep problems. In adulthood, she reported worsened anxiety and sleep problems, and some depressive symptoms. At the final assessment, she was attending university and living independently.

In summary, the individual case studies were heterogeneous in terms of symptomatology, impairment and service use. Both classes showed impairment and some symptomatology across development, but the key differences were an earlier onset of symptoms, more severe symptomatology and greater impairment and service use in the childhood-emerging class. However, results also serve to emphasise the uniqueness of individuals within the trajectory classes.

## 4 Discussion

Using data from a longitudinal study of young people at high-familial risk of depression, we characterized depression trajectories, examined predictors and briefly described several individuals from each class. We identified two distinctive depression trajectory classes: an adulthood-emerging class and a childhood-emerging class. The childhood-emerging class was associated with lower IQ, higher ADHD symptoms, parental depression of a greater severity and more parental comorbidities. Although functional impairment was greater in the childhood-emerging class, both classes showed scores that indicated impaired functioning. Descriptions of the childhood-emerging class indicated severe impairment and symptomatology and high rates of service use. Those of the adulthood-emerging class indicated mixed symptomatology of a subthreshold level and lower levels of impairment compared to the childhood-emerging class.

The two trajectory classes differed considerably in the age at which risk of broadly defined depressive disorder became elevated (defined as a probability of 0.5 or higher). The childhood-emerging class reached this point at age 12.5 and the adulthood-emerging class at age 26. The childhood-emerging group is comparable in some ways to early-onset depression trajectory classes found in the general population, which showed significant risk of clinically elevated depressive symptoms by age 13 (Rice et al., 2019; Weavers et al., 2021). However, as expected given this study focused on the children of depressed parents, there was a noticeably greater proportion of individuals who fell into the childhood-emerging class (25 %) in this sample, compared to general population samples, where usually <10 % report early-onset and persistent symptoms (Musliner et al., 2016). This is despite using a more stringent cut-point for defining depression in the current study compared to self-reported depression scores commonly used in general population studies. The adulthood-emerging class may reflect a more ‘typical’ age of onset for depressive disorders, as early-adulthood is described as a high-risk period for the onset of depression in the general population (Patton et al., 2014; Solmi et al., 2021). Nonetheless, 75 % of this high-familial risk sample fell into the adulthood-emerging class which is substantially higher than what has been reported in general population trajectory studies, which generally report rates of around 25 % or lower (Eaton et al., 2008; Kwong et al., 2019; Schubert et al., 2017; Weavers et al., 2021).

Another notable finding is that unlike in studies of the general population, we did not identify a ‘persistently low depression’ class. The majority of general population samples fall into a class that shows consistently low depressive symptoms (Musliner et al., 2016; Schubert et al., 2017), however we found here that the majority of individuals fell into the adulthood-emerging class. This observation is consistent with elevated rates of depressive symptoms and disorder seen in high-risk samples (Powell et al., n.d.; Weissman et al., 2006). Furthermore, a previous study of this cohort found that 1 in 5 showed sustained good mental health when followed up to adolescence (Collishaw et al., 2016), however no study has yet followed the cohort into early adulthood. It is possible that we do not find a ‘low depression’ class because rates of mental health resilience decrease over time. Indeed, given that the peak period of onset for depressive disorder is in early adult life (Solmi et al., 2021) it is possible that mood-resilience may reduce during this period. It was also somewhat surprising that we did not find a trajectory class where depressive symptoms emerged in adolescence. However, a three-class solution (Supplementary Fig. S3) did identify an adolescent-emerging group (52 %) that showed increasing levels of depressive disorder during adolescence that continued to increase into early adulthood. Thus, it is possible that due to the small sample size and heterogeneity within classes we were unable to identify a distinct adolescent-emerging class or a class with persistently low or no depression. Clinical descriptions of both trajectory classes were variable across individuals, which is consistent with heterogeneity within classes.

Over 70 % of the childhood-emerging class had a parent who had previously experienced a severely impairing episode of depression. Although it is already well-established that severe parental depression is associated with increased rates of depression in offspring (Mars et al., 2012), our results are consistent with previous research which found that children of depressed parents show not only increased rates of depression, but a range of other difficulties compared to children who do not have a depressed parent (Jaffee et al., 2021; Weissman et al., 1987). Familial loading is also not limited to MDD (Rice et al., 2002), and although not significantly different across classes, it was interesting that the polygenic scores were consistently higher in the childhood-emerging class. However, this should be interpreted with caution due to the small sample size and would benefit from future follow-up in larger high-risk studies.

Limitations of the study were attrition at the final assessment, predicted by low family income, single parent household status and parents not having formal secondary school qualifications (Powell et al., n.d.). This might mean that results of this study are less likely to be generalisable to those in low income, single parent households and of lower parental education level. These are also risk factors for offspring depression (Cadman et al., 2021; Giannelis et al., 2021; Torvik et al., 2020) which could mean our estimates of depression in early adulthood are conservative. Of note, prior depression and depression severity in the parent or child did not predict attrition, suggesting that participants who were more unwell were not more likely to drop out, a pattern typically seen in the general population (Rice et al., 2019). The nature of accelerated models meant that we were unable to look at outcomes of trajectory class and were limited to predictors that preceded the study period. The majority of the parents who took part in the study were mothers, so results may not generalise to fathers. Due to the small sample size, the genetic analysis is likely to be underpowered and therefore results need to be interpreted with caution and repeated with larger high-familial risk samples.

Strengths of this study included the use of a large cohort of children of depressed parents followed up for 13 years, which used clinically relevant measures from multiple informants. Clinical reports provided a useful adjunct to quantitative results to describe the trajectory classes. By using a broadly defined depressive disorder category, we overcame a criticism of previous work, whereby the use of self-reported questionnaire measures that do not assess impairment meant that the clinical relevance of such measures was unclear (Musliner et al., 2016). By focusing on a broadly defined depressive disorder category we were able to capture individuals who were impaired by depressive symptoms (Angold et al., 1999b).

Our study demonstrates that the course of depressive disorder in children of depressed parents is variable, with some individuals showing severe persistent depression and others not showing significant symptomatology until early-adulthood. Depression with an earlier onset was associated with lower IQ, higher ADHD traits and parental depression of a greater severity. Crucially, nearly all individuals showed some functional impairment across the study period, differentiating this sample from the general population. This study highlighted that young people at high-risk of depression, particularly those with early-onset and persistent symptoms, should be targets for early intervention strategies for a range of impairing difficulties.

## Funding

The work was supported by the 10.13039/501100000265Medical Research Council (MR/R004609/1) and The Wolfson Centre for Young People's Mental Health, established with support from the 10.13039/501100001320Wolfson Foundation. The cohort was established with funding from the 10.13039/501100000282Jules Thorn Charitable Trust (JTA/06). The fourth wave of data collection was funded by 10.13039/501100007155Medical Research Council (MR/R004609/1).

## CRediT authorship contribution statement

BW, FR, LR and JH contributed to the study concept and design. BW, LR, FR, JH and JM contributed to the statistical analysis and RA did the analysis of genetic data. BW, FR and LR drafted the manuscript and all authors critically revised the manuscript. The corresponding author had final responsibility for the decision to submit for publication.

## Declaration of competing interest

Authors BW, LR, JM, RA, SC, JH, AKJ, AT and FR declare that they have no known competing financial interests or personal relationships that could have appeared to influence the work reported in this paper.

## Appendix A Supplementary data

Supplementary material Image 1
