
JOURNAL INFORMATION
==============================
NLM Title Abbreviation: J Affect Disord
Journal ID: J Affect Disord
NLM Journal ID: 7906073

Journal of affective disorders

ISSN: 0165-0327
EISSN: 1573-2517

ARTICLE INFORMATION
==============================
PMCID: PMC7615675
Article ID: EMS193659
PMID: 37394315
DOI: 10.1016/j.jad.2023.06.039
Article ID: EMS193659
Article ID: WTPA193659
Article ID: WTMS193659
Article ID: UKMS193659
Article version: 358
Subjects: Article

Corrigendum to Characterising depression trajectories in young people at high familial risk of depression

Weavers Bryony a b
Riglin Lucy a b
Martin Joanna a b
Anney Richard b
Collishaw Stephan a b
Heron Jon c
Thapar Ajay a b
Thapar Anita a b
Rice Frances a b
a Wolfson Centre for Young People’s Mental Health, Division of Psychological Medicine and Clinical Neurosciences, Cardiff University, Wales, UK
b Centre for Neuropsychiatric Genetics and Genomics, Division of Psychological Medicine and Clinical Neurosciences, Cardiff University, Wales, UK
c Population Health Sciences, Bristol Medical School, University of Bristol, Bristol, Gloucestershire, UK
Bryony Weavers: Weaversb1@cardiff.ac.uk
Print publication date: 2023 Oct 15
Electronic publication date: 2023 Jul 1
Volume: 339
First page: 1002
Last page: 1002
Author manuscript; available in PMC: 2024 Mar 1

License: This work is licensed under a BY 4.0 International license.
License URL: https://creativecommons.org/licenses/by/4.0/

Erratum for:
Erratum for: Characterising depression trajectories in young people at high familial risk of depression 337 15 9 2023 66 74 Journal of Affective Disorders PMC10824668 37224886

==============================
Data Access Statement

Due to ethical restrictions, data collected at assessment waves 1 to 3 cannot be made openly available. Supporting data collected at assessment wave 4 is openly available from the Cardiff University data repository at http://doi.org/10.17035/d.2023.0263728184.

The authors would like to add in a data access statement for the data used in the analysis.

