# Supplementary material for: Characterising depression trajectories in young people at high familial risk of depression
Source: J Affect Disord. Author manuscript; Available in PMC 2024 Feb 1. (PMC10824668; doi:10.1016/j.jad.2023.05.063)
Supplement: Supplementary [file EMS193641-supplement-Supplementary.docx]

**Supplementary Materials**

Supplementary Text

*Data collection after March 2020*

Three parent interviews were conducted between March 2020 and September 2020. These were conducted remotely, via telephone or video calls, due to the COVID-19 pandemic restrictions.

*Validators of trajectory class*

DSM-IV (American Psychiatric Association, 1994) diagnosis of Major Depressive Disorder (MDD) were derived using the semi-structured interviews done at each assessment point. Tick lists of DSM-IV MDD criteria were completed based on parent- and self-reports of depressive symptoms and associated impairment in the Child and Adolescent Psychiatric Assessment (CAPA) (Angold, Prendergast, et al., 1995) and its adult extension, The Young Adult Psychiatric Assessment (YAPA) (Angold et al., 1999). If a young person met criteria for MDD or showed subthreshold symptoms, their interview was taken to a clinical consensus meeting where psychiatric diagnoses were discussed and agreed upon by two psychiatrists. Depressive symptoms were measured using the Mood and Feelings Questionnaire (MFQ) (Angold, Costello, et al., 1995). The self-reported MFQ has 33 items and the parent reported MFQ has 34 items. All items are scored as not true = 0, sometimes true = 1 and true = 2. A total score for each report is calculated by summing all items (total score 0-66 or 0-68). The MFQ has been shown to be a valid measure of depression, with scores of 27 or more on the self-reported questionnaire, and a scores of 21 or more in the parent-reported questionnaire indicating the presence of MDD (Wood et al., 1995). The MFQ has a high sensitivity and specificity for discriminating those with and without MDD in both children and young adults (Eyre et al., 2021; Wood et al., 1995). Functional impairment was measured using the Strength and Difficulties Questionnaire (SDQ) impact supplement (Goodman, 1999). This comprises five questions asking about impairment in four domains: home life, friendships, studies or work, and leisure activities. These are scored on a four-point scale, where not at all = 0, only a little = 1, quite a lot = 2 and a great deal = 3. A total score of 2 or more (range 0 – 10) is considered as an indication of impairment (Goodman, 1999).

*Polygenic scores*

*Genotyping*

DNA samples were collected during study assessments via saliva samples using Oragene sample pots from the parent and the young person. Raw genotypes were generated using the InfiniumPsycharray. Raw genotype data was harmonised to build hg19 and SNP nomenclature was updated to match the Haplotype Reference Consortium r1.1 (McCarthy et al., 2016). All allele mismatches were dropped.

*Genotype imputation*

To improve genotype coverage, genotypes were imputed using the Michigan Imputation Server (https://imputationserver.sph.umich.edu/) in array-matched batches as described in (Lynham et al., 2023). Prior to imputation SNPs with low call rates (> 5% missing), individuals with low genotyping rates (> 5% missing), SNPs with deviation from Hardy-Weinberg Equilibrium (p ≤ 10-6) and those with a minor allele frequency (MAF) < 0.01% were excluded. Imputation was limited to autosomal variation. Imputation was performed with Eagle v2.3 pre-phasing and MiniMac imputation using Haplotype Reference Consortium r1.1 as the reference panel. Post-imputation markers were excluded based on imputation quality score (info<0.8), individual marker missingness (>2%), low minor allele count (MAC<5), deviations from Hardy-Weinberg equilibrium (p ≤ 10-10) and the deviations from the expected minor allele frequency (defined as greater than 4 standard deviations (SD) from GBR MAF reported in 1000G phase 3). Individuals were additionally removed if they showed low genotyping call rate (>2%) and if there was evidence of excessive heterozygosity (defined as greater than 4 SD from sample mean).

*Ancestry matching*

Analysis was limited to individuals who were shown to have similar genotype profile as the GBR reference dataset from the 1000 genomes project. Ancestry-matching was defined as an individual whose first 3 principal components were within 4 standard deviations from the GBR reference population.

*Generating polygenic scores from training GWAS*

Polygenic scores were calculated for 10 P-value thresholds (p < .5, p < .1, p < .05, p < .01, p < .001, p < .0001, p < 1 x 10-5, p < 1 x 10-6, p < 1 x 10-7, p < 1 x 10-8) for the Psychiatric Genomics Consortium GWAS for ADHD (Demontis et al., 2019; n = 20 183 cases and 35 191 controls), bipolar disorder (Mullins et al., 2021; n = 41917 cases and 371549 controls), major depressive disorder (Wray et al., 2018; n = 130664 cases and 330470 controls) and schizophrenia (The Schizophrenia Working Group of the Psychiatric Genomics Consortium et al., 2020; n = 69369 cases and 236642 controls). Prior to calculating scores, training GWAS were harmonised to hp19 and nomenclature standardised to the 1000 genomes reference panel based on chromosome location. Risk alleles were defined as those associated with case status and all association data was limited to approximate linkage equilibrium (clumped with maximum pair-wise LD (r2) of 0.2). Scores were calculated as the sum of the weighted number of risk alleles for the association threshold.

*PGS-PCA*

The PGS-PCA approach involves computing PRS under a range of parameter settings, performing a principal component analysis (PCA) on the resulting set of PRSs and using the first principal component (PC) in association tests. The first PC reweights the variants included in the PRS to achieve maximum variation over all PRS settings used. This method has been shown to outperform other strategies for using PRS in most scenarios (Coombes et al., 2020). We used the PGS-PCA approach with the 10 p-value threshold settings listed above and used the first PGS-PC in association tests. We also included the first five PCs as covariates in the model.

*Clinical descriptions*

Purposeful sampling was used to select individuals for clinical descriptions. Individuals with the highest probability of being assigned to each of the trajectory classes were chosen in order of probability. If the sex of the chosen cases was homogenous then the final exemplar was replaced with the case of the opposite sex who had the next highest probability. This was to ensure that both male and female sex was represented in each trajectory class. Where the class appeared heterogeneous, another exemplar (of the next highest probability of class assignment) was chosen to help clarify the description of that trajectory class. Four clinical exemplars with the highest probability of being in their assigned trajectory class were chosen from each of the two trajectory classes. The fourth case in the childhood-emerging class was replaced due to the originally selected cases being homogenous in sex (all female). It was replaced with the next highest probability case who was of male sex. The four cases with the highest probability were also chosen for the adulthood-emerging class, and in addition another female case was chosen. This was because marked sex differences emerged in the one female selected case compared to the three selected males cases, and it was uncertain whether these were generalisable to the whole trajectory class or specific to that particular female individual.

Supplementary Tables

**Table S1.** Sample size for each age following restructuring of data into an accelerated design format.

| **Age** | **N** |
| --- | --- |
| 9 | 23 |
| 10 | 54 |
| 11 | 97 |
| 12 | 120 |
| 13 | 145 |
| 14 | 136 |
| 15 | 120 |
| 16 | 90 |
| 17 | 55 |
| 18 | 34 |
| 19 | 11 |
| 20 | 7 |
| 21 | 11 |
| 22 | 29 |
| 23 | 23 |
| 24 | 20 |
| 25 | 12 |
| 26 | 18 |
| 27 | 8 |
| 28 | 5 |

Footnote: due to inclusion criteria and timing of assessment waves, sample sizes are smaller at the extreme ends of each assessment wave.

**Table S2.** Age at each wave as a correlate of trajectory class membership.

|  | **Childhood-emerging depression, 24.6%**  **Percentage or mean (S.E)** | **Adulthood-emerging depression, 75.4%**  **Percentage or mean (S.E)** | **Chi-square (p value)** |
| --- | --- | --- | --- |
| **Age at wave 1** | 12.90 (0.274) | 12.23 (0.142) | 3.82 (.051) |
| **Age at wave 2** | 14.26 (0.279) | 13.60 (0.142) | 3.58 (.058) |
| **Age at wave 3** | 15.37 (0.281) | 14.71 (0.148) | 3.57 (.059) |
| **Age at wave 4** | 24.20 (0.306) | 23.37 (0.147) | 4.89 (.027) |

**Table S3.** Testing age and wave effects on meeting criteria for broadly defined depressive disorder.

| **Model** | **Standardised Coefficients Beta** | **t** | **Significance** |
| --- | --- | --- | --- |
| **(Constant)** |  | 2.90 | .004 |
| **Wave** | -.17 | -1.43 | .153 |
| **Child age** | .21 | 1.84 | .066 |
| **Wave * child age** | .24 | 1.17 | .243 |

**Table S4.** Model fit indices of tested trajectory models.

| **Model** | **Linear** | **Quadratic** | **Linear piecewise** | **Quadratic piece 1** | **Quadratic piece 2** | **Quadratic piecewise** |
| --- | --- | --- | --- | --- | --- | --- |
| **SABIC** | 984.043 | 986.021 | 985.732 | 987.579 | 987.419 | 989.642 |
| **LL** | -489.376 | -489.043 | -488.898 | -488.500 | -488.420 | -488.208 |
| **Intercept** | 0 | 0 | 0 | 0 | 0 | 0 |
| **Mean of slope (S1)** | 1.582 (sd=.181)  P<.0001 | 2.152 (sd=.735)  P=.003 | 1.961 (sd=.474)  P<.0001 | 3.690 (sd=2.179)  P=.090 | 2.087 (sd=.524)  P<.0001 | 3.368 (sd=2.186)  P=.123 |
| **Mean of slope (S2)** | - | - | 1.303 (sd=.328)  P<.0001 | 1.450 (sd=.369)  P<.0001 | 0.222 (sd=1.236)  P=.857 | 0.536 (sd=1.367)  P=.695 |
| **Mean of quadratic (Q1)** | - | -0.314 (sd=.380)  P=.409 | - | -1.787 (sd=2.143)  P=.404 | - | -1.347 (sd=2.224)  P=.545 |
| **Mean of quadratic (Q2)** | - | - | - | - | 1.290 (sd=1.369)  P=.346 | 1.047 (sd=1.446)  P=.469 |

Footnote to Table S4. The linear model is a single slope model. The quadratic model tests the possibility of quadratic effects whereby the slope is not linear. Piecewise models allow for age effects on the slope and the intercept due to differing risk of depression at different developmental periods. The piecewise models were split into a younger (age 9 – 17 years) and an older (18 – 28 years) spline to represent the developmental difference between adolescence and early adulthood, and also the transition from child mental health services to adult services.

**Table S5.** Sensitivity analysis excluding irritability.

| **Proportion of individuals meeting broadly defined depression** | | |
| --- | --- | --- |
|  | **Irritability included** | **Irritability excluded** |
| **Wave 1** | 50 (15.1%) | 50 (15.1%) |
| **Wave 2** | 43 (15.6%) | 39 (14.2%) |
| **Wave 3** | 52 (19.0%) | 46 (16.8%) |
| **Wave 4** | 68 (48.6%) | 65 (46.1%) |

Footnote to Table S5. Irritability was excluded as a core symptom and also as a symptom that could contribute to ‘one or more other symptoms’ in the diagnosis of broadly defined depressive disorder.

|  | **R (P value)** | **N** |
| --- | --- | --- |
| **Wave 1** | 0.49 (<.0001) | 336 |
| **Wave 2** | 0.35 (<.0001) | 277 |
| **Wave 3** | 0.47 (<.0001) | 271 |
| **Wave 4** | 0.50 (<.0001) | 143 |

**Table S6**. Pearson Bivariate correlations between parent-rated and self-rated depression symptoms.

**Table S7.** Model fit for one to three class solutions when wave 4 depression is self-report.

| **Number of classes** | **1** | **2** | **3** |
| --- | --- | --- | --- |
| **SABIC** | 974.250 | 912.869 | 916.363 |
| **LL** | -484.480 | -449.822 | -447.602 |
| **Number of parameters** | 2 | 5 | 8 |
| **Entropy** | - | 0.612 | 0.426 |
| **Smallest class** | - | 26% (n=89) | 20% (n=67) |
| **Average latent class probabilities for most likely class membership** | - | Class 1: 0.826  Class 2: 0.918 | Class 1: 0.618  Class 2: 0.815  Class 3: 0.640 |
| **VLMR-LRT p value** | - | <.00001 | .0272 |
| **BLRT p value** | - | <.00001 | .3333 |

Footnote to Table S7. At the fourth wave there were 27 families where the parent took part, but the child did not, therefore a reduced sample size when only including self-reported data at wave 4.

Supplementary Figures

**Figure S1.** Family response rates for each wave.

**Wave 1**: 337 families (recurrently depressed adults and their children) recruited that meet inclusion criteria at baseline

**Wave 2**: 319 families retained (94.7% of baseline sample)

**Wave 3**: 309 families retained (91.7% of baseline sample)

*Average length of follow up = 16 months*

*Average length of follow up = 13 months*

***73 families of the baseline sample (337) were not contactable at wave 4:***

*41 withdrew before wave 4*

*19 withdrew during wave 4*

*4 were lost to follow up / no working contact details*

*9 did not wish to participate this time round (due to illness, bereavement or busy work schedules) but consented to future contact*

**Transition period from adolescence to adulthood**

*Average length of follow up = 8 years*

**Wave 4**: 197 families retained (58.5% of baseline sample; *74.6% of the 264 families who were contactable for participation at wave 4*)

Footnote to Figure S1. The Early Prediction of Adolescent Depression (EPAD) study was conducted between April 2007 and September 2020 in four assessment waves. Families who participated at each wave via questionnaire, interview or both are reported. Only 264 families were contactable at wave 4 due to loss of up-to-date contact details, withdrawal from the study, death and declining to participate due to ill health, bereavement or other commitments such as work (n=73). Of the 264 contactable families at wave 4, 67 were unresponsive despite multiple communication attempts. Of these 67, 48 families were contacted 3 or more times with no response, 2 of the parents had died and there was no response from the offspring, and 17 families initially responded to contact but ultimately did not commit to taking part. Of the 17 who provided details, the most common reason for being unable to commit to taking part was being too busy, namely with work or life events (e.g. moving house) (Powell et al., under review).

**
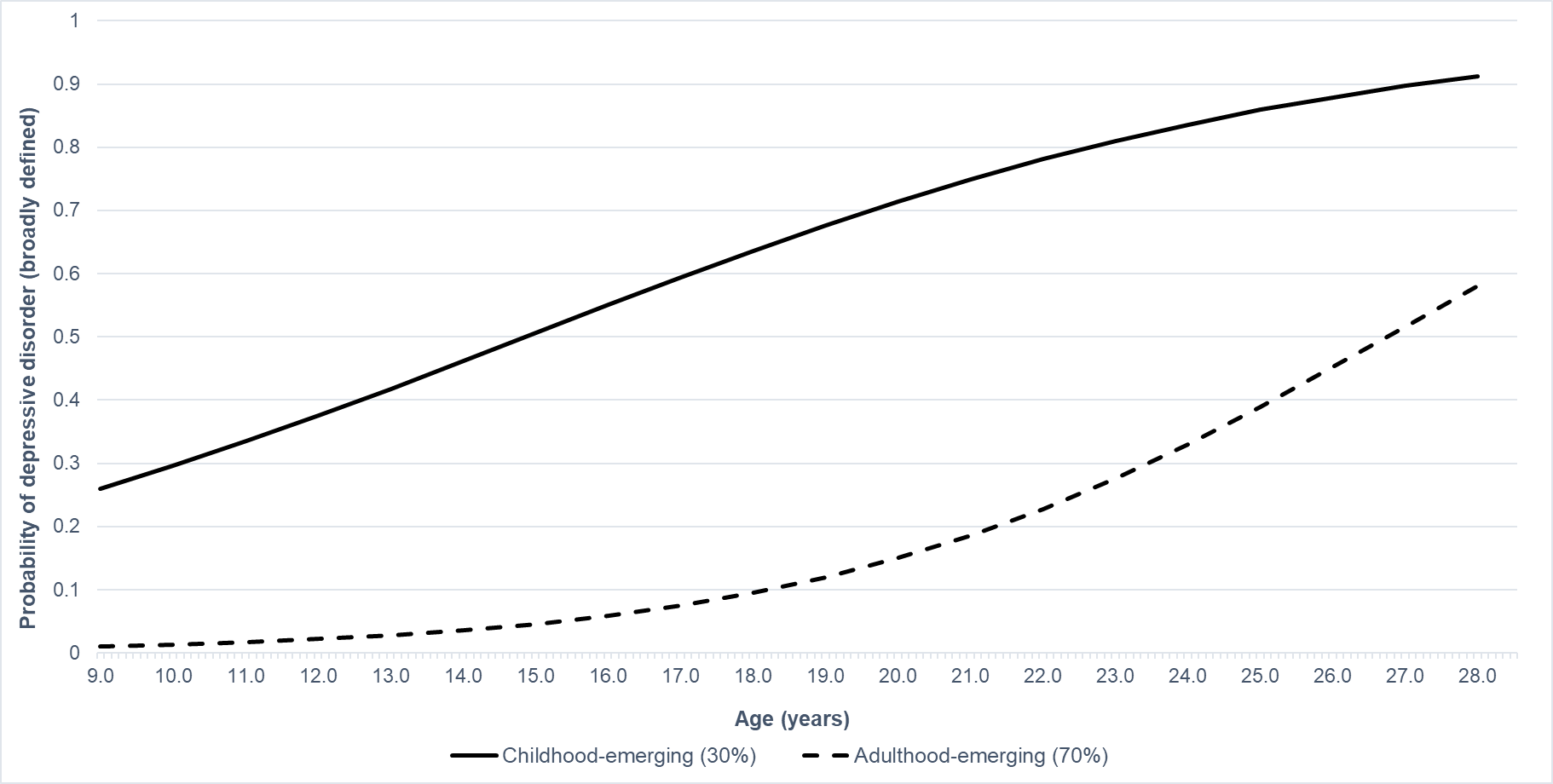
Figure S2.** Trajectory classes when irritability was excluded as a symptom

**Figure S3.** Three class trajectory model

**
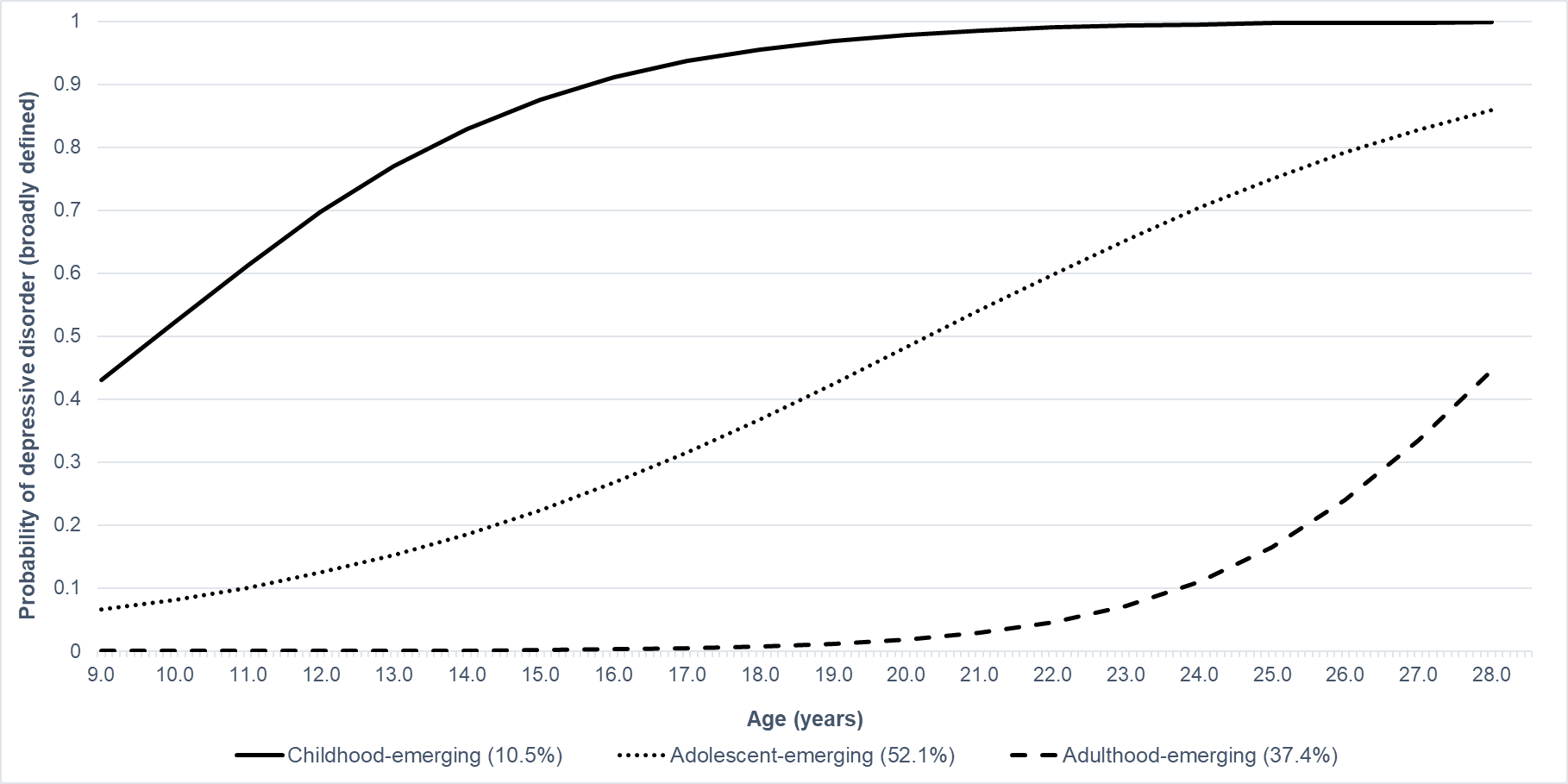
**

References

American Psychiatric Association. (1994). Diagnostic and Statistical Manual of Mental Disorders (4th ed.). *Washington, DC*, 886.

Angold, A., Costello, E., … S. M.-I. journal of, & 1995, U. (1995). Development of a short questionnaire for use in epidemiological studies of depression in children and adolescents. *International Journal of Methods in Psychiatric Research*, *5*(4), 237–249. https://psycnet.apa.org/record/1996-02633-002

Angold, A., Cox, A., Prendergast, M., Rutter, M., Simonoff, E., Costello, E., & Asher, B. H. (1999). *The Young Adult Psychiatric Assessment (YAPA)*. Duke University Medical Center.

Angold, A., Prendergast, M., Cox, A., Harrington, R., Simonoff, E., & Rutter, M. (1995). The Child and Adolescent Psychiatric Assessment (CAPA). *Psychological Medicine*, *25*(4), 739–753. https://doi.org/10.1017/S003329170003498X

Coombes, B. J., Ploner, A., Bergen, S. E., & Biernacka, J. M. (2020). A principal component approach to improve association testing with polygenic risk scores. *Genetic Epidemiology*, *44*(7), 676–686. https://doi.org/10.1002/gepi.22339

Demontis, D., Walters, R. K., Martin, J., Mattheisen, M., Als, T. D., Agerbo, E., Baldursson, G., Belliveau, R., Bybjerg-Grauholm, J., Bækvad-Hansen, M., Cerrato, F., Chambert, K., Churchhouse, C., Dumont, A., Eriksson, N., Gandal, M., Goldstein, J. I., Grasby, K. L., Grove, J., … Neale, B. M. (2019). Discovery of the first genome-wide significant risk loci for attention deficit/hyperactivity disorder. *Nature Genetics*, *51*(1), 63–75. https://doi.org/10.1038/S41588-018-0269-7

Eyre, O., Bevan, R., Ab, J., Shameem, S., Ab, A., Wootton, R. E., Thapar, A. K., Stergiakouli, E., Langley, K., Collishaw, S., Thapar, A., & Riglin, L. (2021). Validation of the short Mood and Feelings Questionnaire in young adulthood. *Journal of Affective Disorders*. https://doi.org/10.1101/2021.01.22.21250311

Goodman, R. (1999). The Extended Version of the Strengths and Difficulties Questionnaire as a Guide to Child Psychiatric Caseness and Consequent Burden. *Journal of Child Psychology and Psychiatry*, *40*(5), 791–799. https://doi.org/10.1111/1469-7610.00494

Lynham, A. J., Knott, S., Underwood, J. F. G., Hubbard, L., Agha, S. S., Bisson, J. I., Bree, M. B. M. van den, Chawner, S. J. R. A., Craddock, N., O’Donovan, M., Jones, I. R., Kirov, G., Langley, K., Martin, J., Rice, F., Roberts, N. P., Thapar, A., Anney, R., Owen, M. J., … Walters, J. T. R. (2023). DRAGON-Data: a platform and protocol for integrating genomic and phenotypic data across large psychiatric cohorts. *BJPsych Open*, *9*(2), e32. https://doi.org/10.1192/BJO.2022.636

McCarthy, S., Das, S., Kretzschmar, W., Delaneau, O., Wood, A. R., Teumer, A., Kang, H. M., Fuchsberger, C., Danecek, P., Sharp, K., Luo, Y., Sidore, C., Kwong, A., Timpson, N., Koskinen, S., Vrieze, S., Scott, L. J., Zhang, H., Mahajan, A., … Marchini, J. (2016). A reference panel of 64,976 haplotypes for genotype imputation. *Nature Genetics*, *48*(10), 1279–1283. https://doi.org/10.1038/NG.3643

Mullins, N., Forstner, A. J., O’Connell, K. S., Coombes, B., Coleman, J. R. I., Qiao, Z., Als, T. D., Bigdeli, T. B., Børte, S., Bryois, J., Charney, A. W., Drange, O. K., Gandal, M. J., Hagenaars, S. P., Ikeda, M., Kamitaki, N., Kim, M., Krebs, K., Panagiotaropoulou, G., … Andreassen, O. A. (2021). Genome-wide association study of more than 40,000 bipolar disorder cases provides new insights into the underlying biology. *Nature Genetics*, *53*(6), 817–829. https://doi.org/10.1038/S41588-021-00857-4

Powell, V., Lennon, J., Bevan Jones, R., Stephens, A., Weavers, B., Osborn, D., Allardyce, J., Potter, R., Thapar, A., Collishaw, S., Thapar, A., Heron, J., & Rice, F. *Following the children of depressed parents from childhood to adult life: a focus on mood and anxiety disorders*. Manuscript submitted for publication.

The Schizophrenia Working Group of the Psychiatric Genomics Consortium, Ripke, S., Walters, J. T., & O’Donovan, M. C. (2020). Mapping genomic loci prioritises genes and implicates synaptic biology in schizophrenia. *MedRxiv*, 2020.09.12.20192922. https://doi.org/10.1101/2020.09.12.20192922

Wood, A., Kroll, L., & Moore, A. (1995). Properties of the Mood and Feelings Questionnaire in Adolescent Psychiatric Outpatients: A Research Note. *Journal of Child Psychology and Psychiatry*, *36*(2), 327–334. https://doi.org/10.1111/J.1469-7610.1995.TB01828.X

Wray, N. R., Ripke, S., Mattheisen, M., Trzaskowski, M., Byrne, E. M., Abdellaoui, A., Adams, M. J., Agerbo, E., Air, T. M., Andlauer, T. M. F., Bacanu, S. A., Bækvad-Hansen, M., Beekman, A. F. T., Bigdeli, T. B., Binder, E. B., Blackwood, D. R. H., Bryois, J., Buttenschøn, H. N., Bybjerg-Grauholm, J., … Sullivan, P. F. (2018). Genome-wide association analyses identify 44 risk variants and refine the genetic architecture of major depression. *Nature Genetics*, *50*(5), 668–681. https://doi.org/10.1038/s41588-018-0090-3
